# A Rapid UPLC-MS Method for Quantification of Gomisin D in Rat Plasma and Its Application to a Pharmacokinetic and Bioavailability Study

**DOI:** 10.3390/molecules24071403

**Published:** 2019-04-10

**Authors:** Xiaoyong Zheng, Feng Feng, Xiunan Jiang, Jieying Qiu, Xiaojun Cai, Zheng Xiang

**Affiliations:** School of Pharmaceutical Sciences, Wenzhou Medical University, Wenzhou 325035, China; zhengxiaoyong71@163.com (X.Z.); FF000999@163.com (F.F.); 13387667238@163.com (X.J.); qiujieying0109@163.com (J.Q.); xiaocaixj@163.com (X.C.)

**Keywords:** UPLC-MS/MS, gomisin D, pharmacokinetic, bioavailability

## Abstract

Gomisin D, a lignan compound isolated from *Fructus Schisandra*, is a potential antidiabetic and anti-Alzheimer’s agent. Recently, gomisin D was used as a quality marker of some traditional Chinese medicine (TCM) formulas. In this study, a rapid ultra-performance liquid chromatography/tandem mass spectrometry method (UPLC-MS/MS) was developed and validated to quantify gomisin D in rat plasma for a pharmacokinetic and bioavailability study. Acetonitrile was used to precipitate plasma proteins. Separations were performed on a BEH C18 column with a gradient mobile phase comprising of acetonitrile and water (0.1% formic acid). An electrospray ionization source was applied and operated in the positive ion mode. The multiple reaction monitoring mode (MRM) was utilized to quantify gomisin D and nomilin (internal standard, IS) using the transitions of *m*/*z* 531.2 → 383.1 and *m*/*z* 515.3 → 161.0, respectively. The calibration curve was linear over the working range from 1 to 4000 ng/mL (*R^2^* = 0.993). The intra- and interday precision ranged from 1.9% to 12.9%. The extraction recovery of gomisin D was in the range of 79.2–86.3%. The validated UPLC-MS/MS method was then used to obtain the pharmacokinetic characteristics of gomisin D after intravenous (5 mg/kg) and intragastric (50 mg/kg) administration to rats. The bioavailability of gomisin D was 107.6%, indicating that this compound may become a promising intragastrical medication. Our results provided useful information for further preclinical studies on gomisin D.

## 1. Introduction

*Fructus Schisandrae* is a Chinese folk herb with a variety of pharmacological activities, including antihepatotoxic, antihyperlipidemic, antiasthmatic, hypoglycemic, and antigastric ulcer activities [1,2,3,4,5,6,7,8,9]. What is more, *Fructus Schisandrae* is frequently used in combination chemotherapy regimens with other drugs for schizophrenia patients in order to lower side effects and improve therapeutic efficacy [10,11]. Modern pharmacological studies indicate that most of the pharmacological and biological actions of *Fructus Schisandrae* can be attributed to lignans, which account for approximately 1% of the fruits’ composition and consist of over 100 related compounds [12,13]. Gomisin D is a lignan found in *Fructus Schisandra* and has been demonstrated to have an antidiabetic effect and to inhibit of UDP-Glucuronosyltransferases activity [8,14,15]. In addition, gomisin D is able to scavenge ABTS (+) radicals [16] and treat Alzheimer’s disease [17,18,19]. Recently, gomisin D is used as a quality marker of Shengmai San [18] and shenqi Jiangtang Granule [15]. However, even with the hot research on pharmacological activity, there is little information about the pharmacokinetic characteristics and bioavailability of monomer’s gomisin D. Therefore, it is necessary to develop a rapid analytical method for the determination of gomisin D in biological matrix for its further clinical application and pharmacological studies.

Some high-performance liquid chromatography (HPLC) methods had been developed to determine gomisin D in different matrices. Smejkal et al. and Shi et al. determine gomisin D from *Fructus Schisandrae* by HPLC with diode array detector (DAD) [13,20]. Mocan et al. reported that gomisin D was identified and determined from *Schisandra chinensis* (Turcz.) Baill by using LC-DAD-QTOF-MS [16]. Zhang et al. establishs an ultra-high-performance liquid chromatography coupled with mass spectrometry (UPLC-MS/MS) method to evaluate the quality of *Fructus Schisandrae* through simultaneous qualitative and quantitative analysis of lignans [21]. Recently, in order to investigates the pharmacokinetic profiles of seven lignans, Sun et al. developed an HPLC-MS/MS method to determine gomisin D in rat plasma after intragastrical administration of *Schisandra chinensis* extract [19]. The retention time is >15 min and the linear range is 1.95–78.1 ng/mL. The maximum and minimum plasma concentrations of gomisin D are <12 µg/mL and >3 µg/mL, respectively. The concentration ranges are far greater than the linear ranges of this method. Su et al. also developed an UPLC-MS/MS method for the simultaneous determination of 12 lignans in rat plasma after administration of wine-processed Schisandra Chinensis fructus (WPSCF) extract [22]. The retention time of gomisin D is >11 min and the linear range is 5.0–625.0 ng/mL. Obviously, the method with a longer retention time and narrower linear ranges was not suitable for pharmacokinetics of gomisin D. In addition, up to now, no pharmacokinetic study of a monomer of gomisin D has been performed, which hinders its clinical application. In this work, a wider linear and rapid UPLC-MS/MS method was developed to determine gomisin D in rat plasma, and was successfully applied to investigate the pharmacokinetics and bioavailability of gomisin D in rats after i.v. and i.g. administration.

## 2. Experimental

### 2.1. Materials 

**Figure 1:**
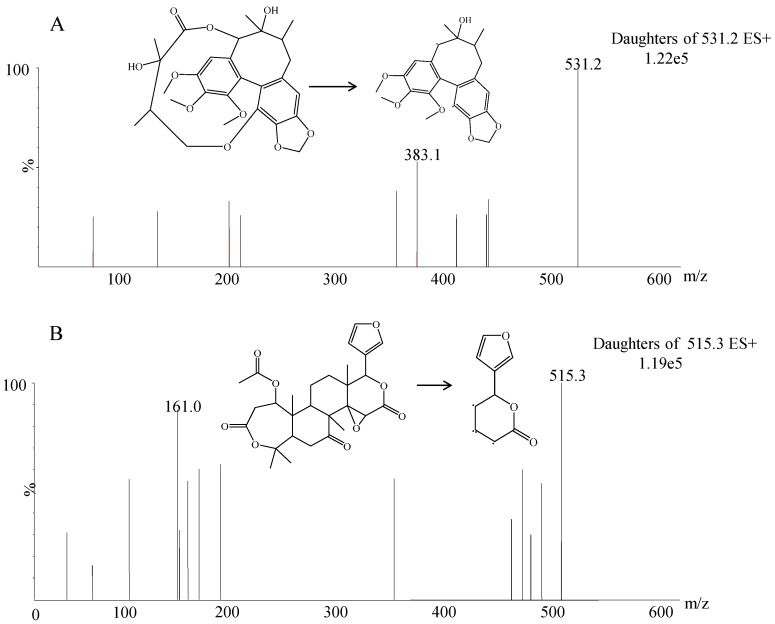
Mass spectra of gomisin D (**A**) and nomilin (**B**) in scan mode with an electrospray ionization (ESI) (+) source.

Gomisin D (purity ≥98%) and nomilin (purity ≥98%) as an internal standard (IS) were purchased from Chengdu Munster Biotechnology Co., Ltd. (Chengdu, China) (Figure 1). Formic acid (Sigma Aldrich, St. Louis, MO, USA), methanol and acetonitrile (Merck, Darmstadt, Germany) were of HPLC grade. Ultrapure water (18.2 mΩ) was prepared from a Milli-Q system (Millipore, Bedford, MA, USA). All other reagents were HPLC or analytical grade.

### 2.2. Instruments and Conditions

All samples were analyzed on a Waters Acquity-UPLC (Water, Milford, MA, USA). The temperature of the autosampler was set at 4 °C. Separations was performed on an ACQUITY UPLC^®^ BEH C18 (2.1 × 50 mm, 1.7 µm) column at temperature of 35 °C. The gradient mobile phases consisted of solvent A (0.1% formic acid) and solvent B (acetonitrile) as follows: (1) held at 10% B (0 min to 0.2 min); (2) increased from 10% to 80% B (0.2 min to 1.5 min); (3) held at 80% B (1.5 min to 2 min); (4) decreased from 80% to 10% B (2 min to 2.2 min); (5) held at 10% B (2.2 min to 4 min). The flow rate and injection volume were 0.4 mL/min and 3 µL, respectively. 

The ESI source was operated in positive ion mode. The optimized mass spectrometer parameters were set as follows: capillary voltage, 1.93 kV; and desolvation temperature, 600 °C. Argon was used as a collision gas. Nitrogen was used as desolvation gas (1000 L/h) and cone gas (50 L/h). The optimal collision energies of gomisin D and IS were 18 and 32 V, respectively. The cone energy was set at 6 V for gomisin D and 35 V for IS. The ion transitions of MRM were *m*/*z* 531.2 → 383.1 and *m*/*z* 515.3 → 161.0 for gomisin D and IS, respectively. 

### 2.3. Preparation of Standard and Quality Control Samples

Stock solutions of gomisin D and nomilin were respectively prepared at a precise concentration of 700.0 µg/mL in methanol and were then serially diluted with methanol to obtain standard working solutions of desired concentrations. Calibration standard solutions were prepared by adding the working solutions into blank rat plasma. Gomisin D was set at concentrations of 1, 2, 5, 10, 20, 50, 100, 200, 500, 1000, and 4000 ng/mL. The quality control (QC) samples with four levels (1, 2, 50, 3200 ng/mL) were prepared by spiking 10 µL of gomisin D working solution into 100 µL of blank rat plasma. All the solutions were stored at 4 °C before use.

### 2.4. Animal Experiments

Twenty-four male Sprague–Dawley rats (250 ± 20 g) were obtained from Laboratory Animal Center of Wenzhou Medical University (Wenzhou, China). All experimental procedures were complied with the “Principles of Laboratory Animal Care” and approved by the Animal Ethics Committee in Wenzhou Medical University (20 July 2018, no: 2018-257). The rats were fasted for 12 h before administration but had free access to water. Twelve rats were intravenously administrated with 5 mg/kg gomisin D, and about 0.25 mL of blood samples were collected at 0.083, 0.25, 0.5, 1, 2, 4, 8, 12, 24 h after administration. Another 12 rats were intragastrically administrated with 50 mg/kg gomisin D, and about 0.25 mL of blood samples were collected at 0.167, 0.333, 0.667, 1, 2, 4, 8, 12, 24, 30 h after administration. All blood samples were taken from rat tail vein and were placed into 1.5 mL heparinized tubes. After centrifugation at 3500 rpm and 4 °C for 10 min, the supernatant was collected and frozen at −20 °C until analysis.

### 2.5. Sample Preparation

The frozen plasma samples were placed at room temperature and thawed before analysis. One hundred μL of plasma sample was firstly transferred to a 1.5 mL polypropylene tube together with 10 μL of IS solution. Then, 290 μL of acetonitrile was added, and vortexed for 2 min to remove protein. After centrifuging at 11,000× *g* for 10 min, the supernatant (80 μL) was carefully transferred to an UPLC vial for analysis.

### 2.6. Method Validation

The validation process of the proposed bioanalytical method complied with the latest guidelines set by the US Food and Drug Administration (FDA) guidelines [23]. The method validation items included selectivity, linearity, precision, accuracy, matrix effect, extraction recovery, and stability.

## 3. Results and Discussion

### 3.1. Method Development

ESI positive and negative modes were both compared and evaluated in method development [24,25,26]. In this work, the response of the positive ion mode showed a better signal-to-noise ratio than that of the negative ion mode. The most abundant fragment ions for MRM was *m*/*z* 531.2 → 383.1 and *m*/*z* 515.3 → 161.0 for gomisin D and IS. The ionic response intensity of *m*/*z* 531.2 → 383.1 was five times higher than that of 531.2 → 401.1 reported in literature [19,22]. In the optimization of UPLC conditions, we found that the mobile phases had an important role in obtaining good chromatographic performances. Acetonitrile and 0.1% formic acid were selected as the mobile phases because they provided sharper peak shapes and shorter retention time. The retention time of 2.29 min was much shorter than that of >11 min reported in literature [19,22]. To find a proper IS, several compounds, including eupatilin, psoralidin, alpinetin, and nomilin, were evaluated. Nomilin was selected as IS because of its similar retention time and appropriate mass response in positive ion ESI mode. Acetonitrile was selected for one-step protein precipitation because it showed a simpler and better effect than other solvents. 

### 3.2. Method Validation

**Figure 2:**
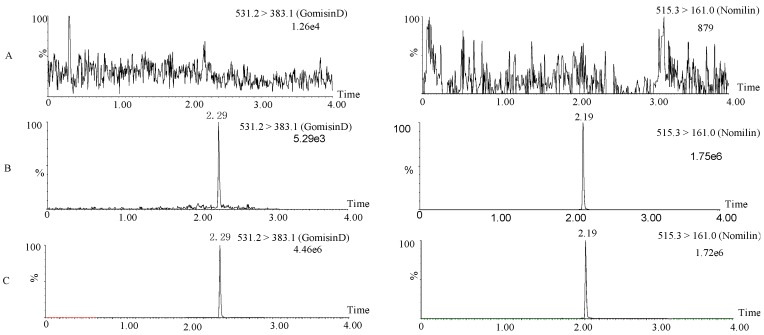
Typical ultra-performance liquid chromatography/tandem mass spectrometry (UPLC-MS) chromatograms of gomisin D and internal standard (IS). Rat blank plasma (**A**); rat blank plasma spiked with 1 ng/mL of gomisin D (**B**); plasma sample collected 30 min after intravenous administration of a single 5 mg/kg dose of gomisin D (**C**).

Selectivity of gomisin D was evaluated at the retention times of the typical chromatograms of blank plasma, blank plasma spiked with gomisin D and IS, and plasma samples after administration. Figure 2 shows there was no significant peak interference at the retention times of gomisin D (2.30 min) and IS (2.19 min), suggesting that no endogenous substances significantly affected the ionization of gomisin D. The calibration curves were linear from 1 to 4000 ng/mL (*R^2^* = 0.993). The LOD and LLOQ were 0.3 ng/mL and 1 ng/mL, respectively. The extraction recovery of gomisin D was greater than 79.2%. The matrix effect of gomisin D was 80.9–86.7%. The detailed extraction recovery and matrix effect of gomisin D are shown in Table 1. To evaluate the precision (RSD) and accuracy (RE) of the method, RSD and RE for QCs at four concentrations were calculated. Table 2 shows that the inter-day RSD ranged from 1.9% to 11.9% and the intraday RSD ranged from 3.3% to 12.9%. The inter- and intraday RE ranged between 85.8% and 98.4% and between 85.4% and 96.6%, respectively. The stability RSD of gomisin D ranged between 1.5% and 10.2% at room temperature for 12 h, at 4 °C for 24 h, at −20 °C for 15 days, and three complete freeze/thaw cycles. The detailed stability RSD of gomisin D was shown in Table 3. The above results demonstrated that these values were acceptable for biological analysis method.

### 3.3. Pharmacokinetic Analysis

**Figure 3:**
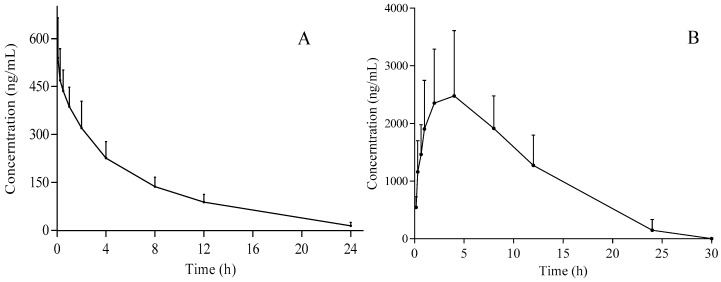
Plasma concentration–time plots of gomisin D after i.v. (**A**) and i.g. (**B**) administration.

The developed UPLC-MS/MS method was applied to the pharmacokinetic and bioavailability study of gomisin D after i.g. and i.v. administrations of 50 and 5 mg/kg, respectively. The mean plasma concentration versus time profiles (*n* = 12) are illustrated in Figure 3. The main pharmacokinetic parameters from noncompartment model analysis are summarized in Table 4. For i.v. administration, T_max_ was 0.083 ± 0.0 h and t_1/2_ was 5.0 ± 1.1 h. The V and CL of gomisin D was 11.2 ± 2.6 L/kg and 1.6 ± 0.3 L/h/kg, respectively. The AUC_(0–t)_ and AUC_(0-∞)_ was 3136.2 ± 548.7 µg L/h and 3261.1 ± 600.0 µg L/h, respectively. For i.g. administration, T_max_ was 3.0 ± 1.2 h and t_1/2_ was 5.6 ± 2.0 h. The V and CL of gomisin D were 13.3 ± 6.2 L/kg and 1.5 ± 0.3 L/h/kg, respectively. The AUC_(0–t)_ and AUC_(0-∞)_ were 32,795.6 ± 11,104.6 µg L/h and 35,091.7 ± 8092.8 µg L/h, respectively. The result of apparent volume of distribution (V) indicated that gomisin D was distributed in extracellular fluid. The results of t_1/2_ showed that gomisin D experienced a slow elimination process after i.v. or i.g. administration, thus possibly obtaining a good therapeutic effect. The bioavailability could reach up to 107.6% as calculated by the formula of F = [(AUC*_i.g._*) × (Dose*_i.v._*)]/[(AUC*_i.v._*) × (Dose *_i.g._*)] × 100%, suggesting that gomisin D may become a promising intragastrical medication. In fact, most lignan compounds show a higher absorption or bioavailability, such as gomisin J [2,27,28]. Gomisin D shows better absorption than other lignans [19]. There were many factors leading to the higher bioavailability (>100%) [29,30], such as enterohepatic circulation, nonspecific binding [31], dose–vehicle effects [32], nonstationary pharmacokinetics [33], nonlinearity in pharmacokinetics [34], and in vivo isomerization [33]. Since there were no double peaks in the pharmacokinetic curve of gomisin D, thus enterohepatic circulation could be excluded. However, the specific reasons for the high bioavailability of gomisin D need to be further explored. Gomisin D is a very promising active monomer for lowering blood sugar [14,15,35]. Our research provided the information of pharmacokinetic and bioavailability of gomisin D. According to the ‘Pubmed Compound’ record, the octanol/water partition coefficient value (XLogP3-AA) of gomisin D is 3.5. In the future, water-soluble groups can be added to the molecular structure of gomisin D to increase its water-solubility.

## 4. Conclusions

Compared to the previous HPLC-MS/MS methods, our UPLC-MS/MS method provided a shorter UPLC run time and wider linear range assay for the quantification of gomisin D in rat plasma. It is the first report on the pharmacokinetics and bioavailability of gomisin D. The pharmacokinetic characteristics of gomisin D will help further the understanding of its pharmacological activity and clinical application.

## Figures and Tables

**Table 1:** Extraction recovery and matrix effect of gomisin D and nomilin in rat plasma (mean ± SD, *n* = 6).

Nominal Concentration (ng/mL)	Extraction Recovery (%, Mean ± SD)	Matrix Effect (%, Mean ± SD)
Gomisin D		
2	79.2 ± 6.4	80.9 ± 4.6
50	86.3 ± 5.2	82.9 ± 8.3
3200	85.1 ± 7.6	86.7 ± 9.3
Nomilin		
400	87.8 ± 2.3	84.0 ± 3.0

**Table 2:** Intra- and interday accuracy and precision for gomisin D in rat quality control (QC) samples.

	Added (ng/mL)	Measured (ng/mL)	Accuracy (%)	Precision (RSD, %)
Interday	1	0.8 ± 0.1	85.8	11.9
(*n* = 6)	2	1.7 ± 0.1	86.4	8.04
	50	55.4 ± 1.8	89.2	3.3
	3200	3217.3 ± 60.6	98.4	1.9
Intraday	1	0.8 ± 0.1	85.4	12.9
(*n* = 6)	2	1.8 ± 0.1	88.7	6.1
	50	54.6 ± 2.6	91.7	4.8
	3200	3106.7 ± 104.4	96.6	3.3

**Table 3:** Stability of gomisin D in rat plasma (mean ± SD, *n* = 6).

Storage Condition	Added (ng/mL)	Measured (ng/mL)	RSD (%)
Room temperature (for 12 h)	2	1.4 ± 0.1	9.1
	50	37.8 ± 0.6	1.5
	3200	2905.7 ± 63.4	2.2
Autosampler (at 4 °C for 24 h)	2	1.2 ± 0.1	9.8
	50	48.2 ± 2.9	5.9
	3200	2928.3 ± 64.3	2.2
Freeze-thaw (for 24 h)	2	1.4 ± 0.2	10.2
	50	38.4 ± 2.0	5.1
	3200	2952.4 ± 105.2	3.6
Stored at −20 °C (for 15 days)	2	1.3 ± 0.2	12.2
	50	40.8 ± 3.1	7.7
	3200	2928.3 ± 116.9	3.9

**Table 4:** Pharmacokinetic parameters of gomisin D in two groups (mean ± SD, *n* = 12).

Parameters	*i.v.* (5 mg/kg)	*i.g.* (50 mg/kg)
AUC_(0-t)_ (µg/L·h)	3136.2 ± 548.7	32,795.6 ± 11,104.6
AUC_(0-∞)_ (µg/L·h)	3261.1 ± 600.0	35,091.7 ± 8092.8
MRT_(0-t)_ (h)	6.0 ± 1.0	7.8 ± 1.2
MRT_(0-∞)_ (h)	6.9 ± 1.3	10.6 ± 6.6
t_1/2_ (h)	5.0 ± 1.1	5.6 ± 2.0
T_max_ (h)	0.083 ± 0.0	3.0 ± 1.2
CL (L/h/kg)	1.6 ± 0.3	1.5 ± 0.3
V (L/kg)	11.2 ± 2.6	13.3 ± 6.2
C_max_ (µg/L)	539.5 ± 125.5	2575.2 ± 1048.2
F (%)		107.6
